# Heat shock proteins HSP70 and MRJ cooperatively regulate cell adhesion and migration through urokinase receptor

**DOI:** 10.1186/1471-2407-14-639

**Published:** 2014-08-30

**Authors:** Yuli Lin, Nana Peng, Hongqin Zhuang, Di Zhang, Yao Wang, Zi-Chun Hua

**Affiliations:** The State Key Laboratory of Pharmaceutical Biotechnology, Nanjing University, Nanjing, 210093 Jiangsu P.R. China; Changzhou High-Tech Research Institute of Nanjing University and Jiangsu Target Pharma Laboratories Inc, Changzhou, 213164 Jiangsu P.R. China; Division of Critical Care and Surgery, St. George Hospital, University of New South Wales, Sydney, NSW2217 Australia

**Keywords:** Heat shock protein HSP70, MRJ, Association, Urokinase receptor, Cell adhesion, Cell migration

## Abstract

**Background:**

The urokinase-type plasminogen activator receptor (uPAR) is an important regulator of ECM proteolysis, cell-ECM interactions and cell signaling. uPAR and heat shock proteins HSP70 and MRJ (DNAJB6) have been implicated in tumor growth and metastasis. We have reported recently that MRJ (DNAJB6, a heat shock protein) can interact with uPAR and enhance cell adhesion. Here, we identified another heat shock protein HSP70 as a novel uPAR-interacting protein.

**Methods:**

We performed co-immunoprecipitation in human embryonic kidney (HEK) 293 and colon cancer HCT116 cells as well as immunofluorence assays in HEK293 cells stably transfected with uPAR to investigate the association of suPAR with HSP70/MRJ. To understand the biological functions of the triple complex of suPAR/HSP70/MRJ, we determined whether HSP70 and/or MRJ regulated uPAR-mediated cell invasion, migration, adhesion to vitronectin and MAPK pathway in two pair of human tumor cells (uPAR negative HEK293 cells *vs* HEK293 cells stably transfected with uPAR and HCT116 cells stably transfected with antisense-uPAR *vs* HCT116 mock cells transfected with vector only) using transwell assay, wound healing assay, quantitative RT-PCR analyzing mmp2 and mmp9 transcription levels, cell adhesion assay and Western blotting assay.

**Results:**

HSP70 and MRJ formed a triple complex with uPAR and over-expression of MRJ enhanced the interaction between HSP70 and uPAR, while knockdown of MRJ decreased soluble uPAR in HCT116 cells (*P* < 0.05) and reduced the formation of the triple complex, suggesting that MRJ may act as an uPAR-specific adaptor protein to link uPAR to HSP70. Further experiments showed that knockdown of HSP70 and/or MRJ by siRNA inhibited uPAR-mediated cell adhesion to vitronectin as well as suppressed cell invasion and migration. Knockdown of HSP70 and/or MRJ inhibited expression of invasion related genes mmp2 and mmp9. Finally, HSP70 and/or MRJ up-regulated phosphorylation levels of ERK1/2 and FAK suggesting MAPK pathway was involved. All the biological function experiments in cell level showed an additive effect when HSP70 and MRJ were regulated simultaneously indicating their collaborated regulation effects on uPAR.

**Conclusions:**

These findings may offer a novel insight into the interactions between uPAR and HSP70/MRJ and their functions in cell adhesion and migration may provide more understanding of the roles in regulating cancer metastasis.

**Electronic supplementary material:**

The online version of this article (doi:10.1186/1471-2407-14-639) contains supplementary material, which is available to authorized users.

## Background

The urokinase-type plasminogen activator (uPA) receptor (uPAR) and its ligand uPA are involved in numerous physiological and pathological processes including pericellular proteolysis, wound healing, tissue regeneration and tumor progression [1–3]. The uPAR protein belongs to the Ly-6/uPAR/α-neurotoxin protein domain family [4] and is a single chain three-domain glycoprotein designated DI, DII and DIII [5]. Since uPAR is located on the cell surface by a glycosyl phosphatidylinositol (GPI) anchor and lacks a trans-membrane domain, it needs to interact with other partner proteins including integrins to activate cellular signaling pathways [6–8]. There also exist three soluble forms of uPAR, DI, DIDII and DIDIIDIII, which are present in cancer cells, urine, blood and cerebrospinal fluid [9–12]. uPAR expression is up-regulated during inflammation [13] and many other diseases [14] including cancer, and its expression levels correlate with poor prognosis [15–18]. uPA binds to uPAR and converts the zymogen plasminogen into plasmin which promotes degradation of ECM by direct digestion and activation of pro-matrix metalloproteases (MMPs), including MMP-2, -9, -12 and -13 [19]. In addition to the binding of uPA, uPAR initiates signal transduction pathways by interacting with other molecules such as vitronectin, integrins β1/2/3, cytokeratin 8/18 and EGFR (epidermal growth factor receptor) [1, 20]. These interactions with uPAR result in various functional consequences depending on the specific interacting protein. For example, vitronectin binds to uPAR, and once phosphorylated, regulates uPAR-dependent cell adhesion [8, 21, 22]. However, to date, the numerous and varied roles of uPAR in cell adhesion, migration, proliferation, angiogenesis and cancer metastasis are not completely explained by identified known protein interactions. We therefore speculate that there are still additional and as yet unidentified uPAR partners. Recently we have described an uPAR binding protein, heat shock protein MRJ, which can regulate uPAR-mediated cell adhesion to vitronectin [23]. In this paper, we identified another heat shock protein HSP70 which can also interact with uPAR.

Heat shock proteins (HSPs) are a set of highly conserved proteins that are inducible by a wide variety of physiological and environmental factors including anti-cancer chemotherapy, thus allowing the cells to survive to lethal conditions. The HSP70 protein is a member of the DnaK/HSP70 class (HSP701A1, 72KDa) (NM_005345). The MRJ protein is member 6 of the DnaJ/HSP40 homolog subfamily B (DnaJB6) (NM_005494). MRJ is an essential co-chaperone of HSP70, with the N-terminal J-domain necessary for its interaction with HSP70 and its chaperone activity [24, 25]. MRJ drives the substrate specificity of HSP70. They usually cooperate in the binding of proteins at intermediate stages of folding, assembly, and translocation across membranes in almost all cellular compartments. Similar to uPAR, expression levels of HSP70 and MRJ are correlated with metastasis and poor prognosis in breast cancer cells [26]. To date, little is known about interaction of HSP70/MRJ complex with uPAR and the biological significance of HSP70/MRJ complex in regulation of uPAR and its signaling. To answer these questions, we show here that the heat shock proteins HSP70 and MRJ form a triple complex and interact functionally with uPAR to increase uPAR-mediated cell migration and adhesion to vitronectin.

## Methods

### Materials

The vector pRNAT-U6.1/neo was purchased from Genescript, USA. The Plasmid HSP70-PRK5 was kindly provided by Dr. Fei Dow from Beijing Normal University. The anti-ERK1/2 and anti-phospho-ERK1/2 antibodies were purchased from Cell Signaling Technology, anti-FAK, anti-phospho-(pY397) FAK and anti-HSP70 from BD Biosciences, anti-phospho-(pS473) AKT from Epitomics, anti-uPAR, anti-HA, anti-MRJ, anti-flag, anti-tubulin, anti-GAPDH, anti-β-actin, goat-anti mouse HRP IgG and goat anti-rabbit HRP IgG antibodies from Santa Cruz, and anti-Alexa Fluor™-labeled secondary antibodies from Invitrogen.

### Cell culture and transfection

The wild type human embryonic kidney (HEK) 293 and human colon carcinoma (HCT116) and human epithelial cervical cancer Hela cell lines were purchased from the American Type Culture Collection (ATCC, Philadelphia, PA, USA). The HEK293T (HEK 293-uPAR) cells stably transfected with uPAR (HEK 293-uPAR) were kindly provided by Dr. Ying Wei (University of California, San Francisco) [22]. The HCT116 cells stably transfected with antisense-uPAR and HCT116 mock cells (vector-only transfected clone) were generated previously [27]. Cells were all grown in Dulbecco’s modified Eagle’s medium (DMEM) (Hyclone) supplemented with 10% FBS (Hyclone) and 50 U/ml penicillin/streptomycin, and maintained in a humidified atmosphere containing 5% CO_2_ at 37°C. The stable transfected HEK293-uPAR cells, HCT116 mock cells and antisense-uPAR HCT116 cells were cultured in the presence of 0.9 mg/ml and 0.6 mg/ml G418 (Geneticin) to maintain selection, respectively. Cell transfection was performed using Lipofectamine^®^ 2000 transfection reagent (Invitrogen).

### Expression vector construction

The vectors encoding uPAR-HA (HA tag is influenza hem-agglutinin: YDYDVPDYA) and MRJ-flag (flag-tag is a peptide with eight amino acids: DYKDDDDK) were generated by amplifying the human uPAR cDNA and MRJ cDNA and then cloned into the PCI-HA and PRK5-flag vectors respectively [23]. The negative control of uPAR siRNA vector which has the same nucleotide composition but not the same sequence as the uPAR siRNA was cloned into PRNAT-U6.1/neo designated as psiSC-uPAR. The HSP70 siRNA vector (psiHSP70) was constructed previously in our laboratory [28]. The MRJ siRNA insert sequence was designed using siRNA designer software and siRNA Construct Builder software (Genscript). MRJ siRNA was designed and constructed into PRNAT-U6.1/neo using BamHI/HindIII (Takara) and designated as psiMRJ. A negative control for siRNA as a scrambled sequence of the MRJ siRNA target sequence was also cloned into PRNAT-U6.1/neo designated as psiSC.

### Real-time quantitative PCR

Total RNA was isolated from tumor cells with the TRIzol reagent (Invitrogen, Rockville, MD) according to the manufacturer’s instructions. 2 μg of total RNA was subjected to reverse transcription using RevertAid™ First-Strand cDNA Synthesis Kit (Fermentas, Lithuania) with random hexamer primer. 8 μl of the cDNA solution (after 50-100 times dilution) was used for real-time PCR. The genes were amplified in a 20 μl reaction using Power SYBR^®^ Green PCR Master Mix (Applied Biosystems by life technologies, UK) according to the manufacturer’s protocol. The PCR primers were synthesized and shown as: mmp2 upstream: CCGTCGCCCATCATCAA; mmp2 downstream: GGTATTGCACTGCCAACTCTTTG; mmp9 upstream: GGACGATGCCTGCAAGT; mmp9 downstream: ACAAATACAGCTGGTTCCCAATC; upa upstream: GTGGATGTGCCCTGAAGGA; upa downstream: TGCGGATCCAGGGTAAGAAG; upar upstream: GAATGGCCGCCAGTGTTACAG; upar downstream: TGGGCATGTTGGCACATTG; mrj upstream: AAGTGCTGTCGGATGCTAAG; mrj downstream: CCTGAAGACATCATCTGGGT; hsp70 upstream: GGGCCTTTCCAAGATTGCTGT; hsp70 downstream: ATCTCTGCATGTAGAAACCGGAAA. The GAPDH (glyceraldehyde 3-phosphatase dehydrogenase) was used as the reference gene and relative mRNA levels were determined using the 2^-△△Ct^ method. Three independent experiments were performed. Student *t*-test was performed when comparing tumor cells transfected with psiSC, psiMRJ, psiHSP70 and psiMRJ plus psiHSP70 groups. The differences between scramble groups psiSC, psiMRJ, psiHSP70, psiMRJ plus psiHSP70 were significant (*P* < 0.05).

### Co-Immunnoprecipitation (Co-IP) and Western blotting

Transfected and control cells were harvested, washed twice with cold PBS and lysed in lysis buffer (50 mM Tris-HCl (pH 7.4), 250 mM NaCl, 0.5% Triton-X 100, 50 mM NaF, 2 mM EDTA, 1 mM Na_3_VO_4_ and protein inhibitor cocktail) for 45 min. For immunoprecipitation, the lysates were incubated with primary antibody overnight at 4°C, and then with Protein G beads for 2 hours at 4°C. After washing the Protein G beads for 4 times with lysis buffer to eliminate the proteins in nonspecific adsorption, the proteins binding to the beads were subjected to WB analysis. For Western blotting, equal amounts of proteins were subjected to SDS-PAGE and the membrane was incubated for 1 hour in PBS with 0.1% Tween-20 (PBST) containing 5% non-fat skim milk for 1 hour at room temperature. Subsequently, the membrane was incubated for 1 hour at room temperature or 4°C overnight with primary antibody in PBST containing 5% non-fat milk, developed with HRP (horseradish peroxidase)-conjugated goat anti-mouse IgG (Santa Cruz Biotechnology, Santa Cruz, CA, U.S.A.) or anti-rabbit IgG and an ECL^®^ (enhanced chemiluminescence) detection system (Amersham Biosciences, Arlington Heights, IL, U.S.A.), followed by exposure to X-ray film. For loading control, the membranes were stripped and probed with antibodies for GAPDH, α-tubulin or β-actin, as per standard protocols. In addition, the HCT116 mock cells were transfected with selected amount of psiHSP70 or psiMRJ alone or in combination or with 10 μM of the proteasome inhibitor, MG-132 (Sigma), for 8 hours in serum-free media. Cells were lysed and probed with uPAR, HSP70, or MRJ antibody as described above. The blots were also probed with β-actin antibody for comparison.

### Immunofluorescence microscopy

Tumor cells were washed twice in PBS and fixed for 1 hour in cold PBS containing 4% formaldehyde. The cells were washed three times in cold PBS and permeabilized for an additional 1 hour in 0.5% Triton X-100. Non-specific staining was blocked with 3% BSA. Primary antibodies were added for 1 hour at 37°C. After washing with PBS, species specific Alexa Fluor™-labeled secondary antibodies were added for 1 hour at 37°C. After washing with PBS, cover slips were mounted on the glass slides using glycerin. Control experiments in the absence of primary antibodies were run in parallel using the same procedure. Fluorescence microscopy was performed by microscopy (Carl Zeiss Axioplan 2).

### Flow cytometry

Cell-surface uPAR levels were measured by flow cytometry. Treated cells were harvested, washed and resuspended in cold PBS containing 1% BSA (2 × 10^6^ cells/ml). Treated tumor cells were labeled with anti-human uPAR monoclonal antibody for 30 min on ice in the dark and immediately analysed by flow cytometry in APC channel. Suitable negative isotype controls were used to rule out background fluorescence. The data were generated by cytofluorometric analyses of 10,000 events. All data were analysed using CELLQuest software (Becton-Dickinson). For cell apoptosis analysis, cells were transfected with different siRNA vectors. Twenty-four hours later, the ratio of cell apoptosis was determined by flow cytometry analysis as previously described [28].

### Cell cycle analysis

Cells (2 × 10^6^) were treated with psiHSP70 and psiMRJ alone or in combination for 24 hours. Cells were then harvested, washed in PBS, resuspended gently in 5 ml of 100% ethanol, and fixed at 25°C for 1 hour. After washing with PBS, cells were incubated with DNase-free RNase A (200 μg/ml) at 37°C for 1 hour and washed with PBS. PI (10 μg/ml) was added and the cells were incubated at 37°C for 5 min. The distribution of cells with differing DNA content was analyzed on a FACSCalibur flow cytometer with CellQuest software (BD Biosciences, CA, USA) at an excitation wavelength of 530 nm. Fluorescence emission was measured using a 620 nm band pass filter.

### Cell proliferation assay

Cells in the exponential growth phase were seeded into a 96-well plate at a density of 5000 cells per well. After 24 hours, cells were transfected with psiHSP70 and/or psiMRJ. The cells were incubated at 37°C for 24 hours, then the cell viability was determined by the colorimetric MTT [3-(4, 5-dimethylthiazol-2-yl)-2, 5-diphenyl-2H-tetrazolium bromide] assay at wave length 570 nm by TECAN Safire Fluorescence Absorbance and Luminescence Reader (Vienna, VA, USA). The cell viability was calculated according to the formula: Cell viability (%) = average A_570 nm_ of treated group/average A_570 nm_ of control group × 100%. Each experiment was performed in quadruplicate and repeated at least three times.

### F-actin staining assay

After the indicated treatments, cells were fixed with 4% formaldehyde for 1 hour, penetrated with 0.5% Triton X-100 for 1 hour, and then stained with Texas Red-X phalloidin for 1 hour. F-actin was then visualized by microscopy (Carl Zeiss, Axioplan 2).

### Cell adhesion assay

96-well plates were pre-coated with vitronectin (1 μg/ml), or fibronectin (5 μg/ml), or bovine serum albumin (BSA, 10 μg/ml) at 37°C overnight, and the wells were blocked with 1% BSA at 37°C for 1 hour. After washing with DMEM serum-free medium for three times, cells were blocked with 0.1% BSA in DMEM and added to coated plates (5 × 10^3^-1 × 10^4^ cells/100 μl per well). After incubation at 37°C for 1 hour, the plates were washed with PBS for 1-3 times till no cells were left in the BSA coated wells. Adherent cells were fixed with methanol for 10 min at room temperature, stained with Giemsa and quantified by reading absorbance at 550 nm using Safire Fluorescence Absorbance and Luminescence Reader (TECAN). All experiments were repeated at least three times.

### Wound-healing assay

Transfected cells were plated in 12-well culture plates to form cell monolayer (near 70% confluence). After serum starvation for 12 hours, a wound was made with a sterile P-200 micropipette to scrape off the cells. The wells were then washed three times with PBS to remove non-adherent cells and incubated in fresh medium containing FBS. The progress of wound closure was monitored with microphotographs of × 10 magnification taken with light microscope (Carl Zeiss Axioplan 2) at the beginning and the end of the experiments after washing with PBS.

### Transwell assay

To determine cell migration and invasion, transwell assay was carried out using a 24 well cell culture insert with 8 mm pore (3097, Falcon-Becton Dickinson, USA). Transfected cells were cultured in serum-free medium overnight. DMEM containing 20% FBS was used as a chemotactic attractant in lower compartment of the Boyden chamber. Single-cell suspensions were plated at the concentration of 10^5^ cells/ml in 0.5 ml serum-free medium, 1% BSA per well into upper chamber for 24 hours. Cells from the upper surface of the filter were removed with a cotton swab; those underneath were fixed with 4% paraformaldehyde prior to staining with 0.5% crystal violet. Images were captured by 10 × objective lens. Invaded or migrated cells were expressed as the average number of migrated cells per microscopic field over four fields per assay in triplicate experiments.

### Statistical analysis

All data are presented as means ± SD (standard deviation) of at least three independent experiments, each performed at least in triplicate, when normally distributed. The statistical significance of differences was determined by student’s two-tailed *t*-test in 2 groups and one way ANOVA in multiple groups. Statistical differences are presented at probability levels of *P* < 0.05, and *P <* 0.01. All data were analyzed with SPSS 13.0 software.

## Results

### HSP70 was associated with uPAR and formation of the uPAR-HSP70 complex was regulated by MRJ and HSP70

**Figure 1:**
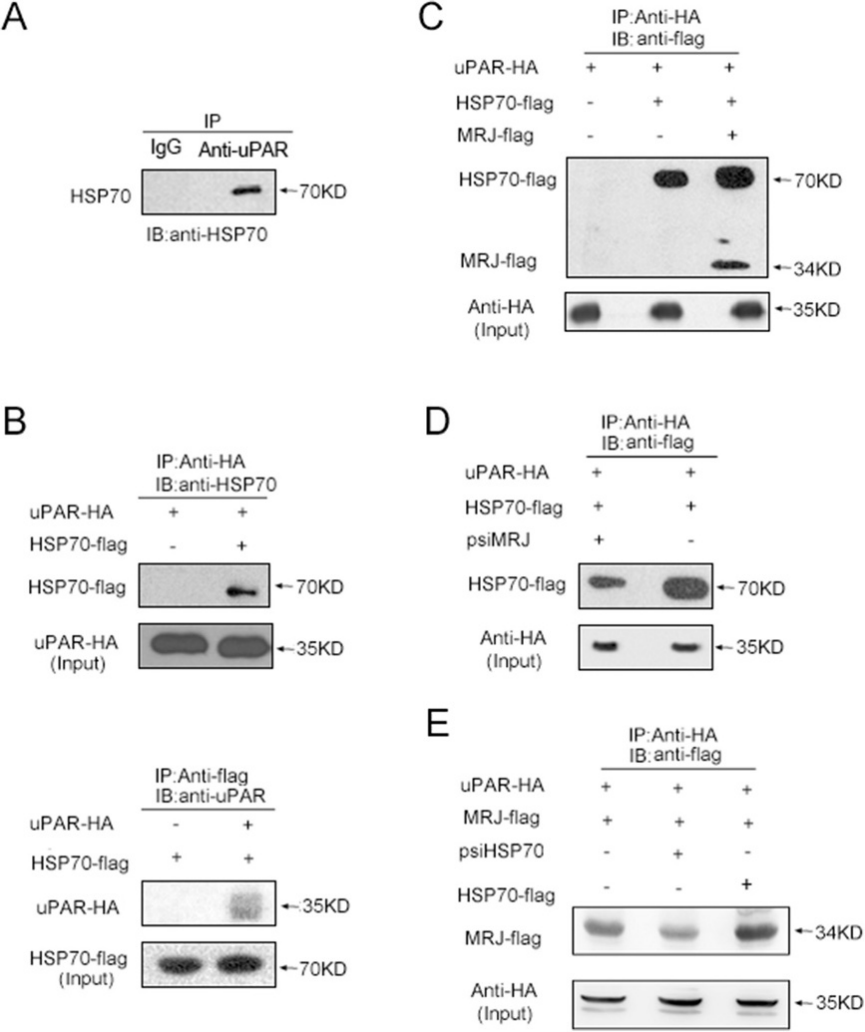
**Association of uPAR with HSP70 and regulation of the uPAR/HSP70 complex by MRJ. (A)** Co-IP of uPAR and HSP70 in HCT116 cells. Cell lysates were immunoprecipitated with anti-uPAR antibody and IgG and the Western blot was probed with anti-HSP70 antibody. **(B)** Co-IP of uPAR and HSP70 in HEK293 cells co-transfected with HSP70-flag and uPAR-HA constructs or transfected with uPAR-HA (top lanes) or HSP70-flag alone (down lanes). IP was performed using anti-HA or anti-flag antibody and WB was performed using anti-flag antibody (upper blot) or anti-HA antibody (lower blot). 6% or 2% of the amount of the original cell lysates used for IP was loaded as an input control and visualized with anti-HA antibody (top lanes) or anti-flag antibody (down lanes), respectively. **(C)** Over-expression of MRJ increased HSP70 in HEK293 cells. The cells were transfected with uPAR-HA, or uPAR-HA plus HSP70-flag or uPAR-HA plus HSP70-flag plus MRJ-flag. IP was performed using anti-HA antibody and WB was then performed using anti-flag to detect the indicated proteins. Equal amounts of protein in each cell lysate were blotted with anti-HA antibody as controls. **(D)** Knockdown of MRJ decreased the HSP70 in HEK293T cells. The cells were co-transfected with uPAR-HA and HSP70-flag, with or without psiMRJ. Co-IP was performed using anti-HA antibody and WB was performed using anti-flag to detect the indicated proteins. Equal amounts of protein in each cell lysate were blotted with anti-HA antibody as controls. **(E)** HSP70 regulated the interaction among HSP70 and MRJ and uPAR. The cells were co-transfected with uPAR-HA plus MRJ-flag, or uPAR-HA plus MRJ-flag plus HSP70-prk5, or uPAR-HA plus MRJ-flag plus psiHSP70. Co-IP was performed using anti-HA antibody and WB was performed using anti-flag to detect the indicated proteins. Equal amounts of protein in each cell lysate were blotted with anti-HA antibody as controls.

We have reported that MRJ can interact with uPAR by a yeast two-hybrid screen, GST-pull down, co-IP and confocal microscopy analyses [23]. Since MRJ has the ability to function together with HSP70, we hypothesized that HSP70 might also interact with uPAR to form a triple complex. To this end, we first determined whether uPAR and HSP70 were associated in wild-type HCT116 cells that express high levels of uPAR. We prepared total lysates from the cells and carried out co-IP and Western blot analyses. As shown in Figure 1A, the HSP70 band was detected in the sample immunoprecipitated with anti-uPAR antibody, but was not detected in the respective IgG isotype controls, indicating a specific interaction between uPAR and HSP70.

To further confirm the interaction between HSP70 and uPAR, vectors expressing epitope tagged HSP70-flag, uPAR-HA and MRJ-flag were generated. Co-IP assays were performed using extracts from HEK293T cells that had been co-transfected with different combinations of these vectors. As shown in Figure 1B, when cells co-transfected with uPAR-HA and HSP70-flag, the HSP70-flag protein was detected in lysate immunoprecipitated with anti-HA (uPAR) antibody, and 35 kDa uPAR-HA protein was detected after immunoprecipitated with anti-flag (HSP70) antibody.

To determinate whether MRJ was involved in the uPAR/HSP70 complex, HEK293T cells were co-transfected with uPAR-HA, or uPAR-HA plus HSP70-flag, or uPAR-HA plus HSP70-flag plus MRJ-flag and then precipitated with anti-HA antibody (Figure 1C). The MRJ-flag protein was detected in lysate immunoprecipitated with anti-HA (uPAR) antibody indicating that uPAR, HSP70 and MRJ formed a triple complex in HEK293T cells. Moreover, the presence of exogenous MRJ or HSP70 could enhance the interaction among HSP70 and MRJ and uPAR. In addition, we found that knockdown of MRJ or HSP70 by its siRNA significantly decreased the interaction in the triple complex in HEK293T cells (Figure 1D and E) suggesting that formation of the complex was regulated by MRJ and HSP70.

### RNAi-mediated down-regulation of HSP70/MRJ reduced uPAR protein level

**Figure 2:**
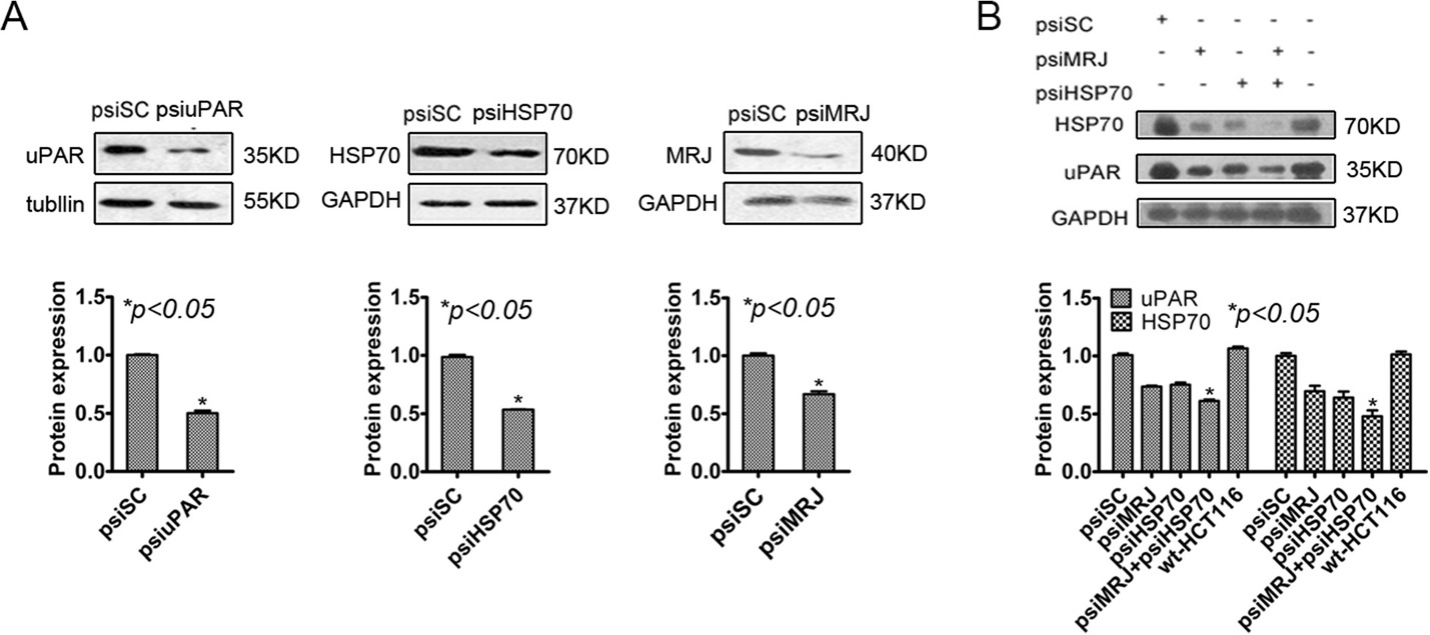
**Inhibition of the soluble uPAR by knockdown of HSP70 and/or MRJ in HCT116 cells. (A)** Effects of siRNAs targeting uPAR, HSP70 and MRJ by Western Blot analysis. The cells were transfected with psiuPAR, or psiHSP70, or psiMRJ or psiSC as shown in the figures. The levels of uPAR, HSP70 and MRJ proteins were normalized to GAPDH levels in psiSC-transfected cells. **(B)** Knockdown of HSP70/MRJ reduced the protein levels of suPAR and HSP70. The cells were transfected with psiSC, or psiMRJ, or psiHSP70, or psiMRJ plus psiHSP70 for 48 hours and processed and then probed with anti-uPAR antibody. The blots were re-probed with GAPDH to confirm equal amounts of protein loading. Protein band intensities were quantified by densitometry analysis using Image J software. Bars indicate the values expressed as the mean ± standard deviation. Statistical evaluation showed that the changes were significant as shown by *P < 0.05. Data shown are representative of three independent experiments.

To confirm the triple complex and to study the regulation of the interaction between MRJ, HSP70 and uPAR, siRNA expressing plasmids targeting uPAR (psiuPAR), MRJ (psiMRJ) and HSP70 (psiHSP70) were constructed and the interference effects were shown in Figure 2A, resulting in 30-60% decreases in their protein levels. We then examined whether suppression of HSP70 and/or MRJ by their siRNA had any effects on protein level of uPAR in the wild type HCT116 cells. The cells were transfected with psiSC, or psiMRJ, or psiHSP70, or psiMRJ plus psiHSP70, and Western blot analyses were performed. As shown in Figure 2B, the expression of uPAR was significantly decreased in cells transfected with psiMRJ, or psiHSP70, or psiMRJ plus psiHSP70 when compared to their psiSC scramble controls (*P* < 0.05). Furthermore, uPAR protein decreased about 20%-30% in HCT116 cells transfected with psiMRJ or psiHSP70, while had about 50% decrease in HCT116 cells transfected with psiMRJ plus psiHSP70 indicating their additive effects (Figure 2B).

**Figure 3:**
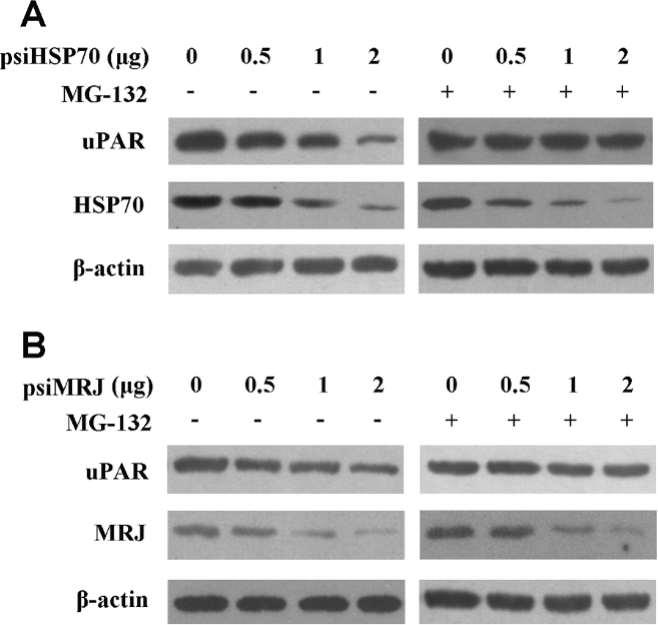
**uPAR is stabilized by HSP70 and MRJ.** Knockdown of HSP70 or MRJ promotes uPAR degradation which is rescued by inhibition of proteasome. The HCT116 mock cells were transfected with the indicated amount of psiHSP70 **(A)** or psiMRJ **(B)** alone or in combination with 10 μM MG-132. Significant degradation is noted for uPAR in the psiHSP70 or psiMRJ only treated cells. The same blot was probed with antibody against β-actin for comparison.

To further characterize the functional interaction of uPAR with HSP70 and MRJ, HCT116 mock cells were transfected with selected amount of psiHSP70 or psiMRJ respectively. As shown in Figure 3, uPAR was shown to be degraded in a dose-dependent manner and this degradation could be rescued by combining the treatment with the proteasome inhibitor MG-132. No significant change in the level of β-actin was noted. These results demonstrate that uPAR is probably a client protein of the HSP70/MRJ chaperone complex and knockdown of HSP70/MRJ prevents stabilization of client protein and leads to the degradation of the client protein by the proteasome.

### HSP70 co-localized with uPAR in the cytoplasm

**Figure 4:**
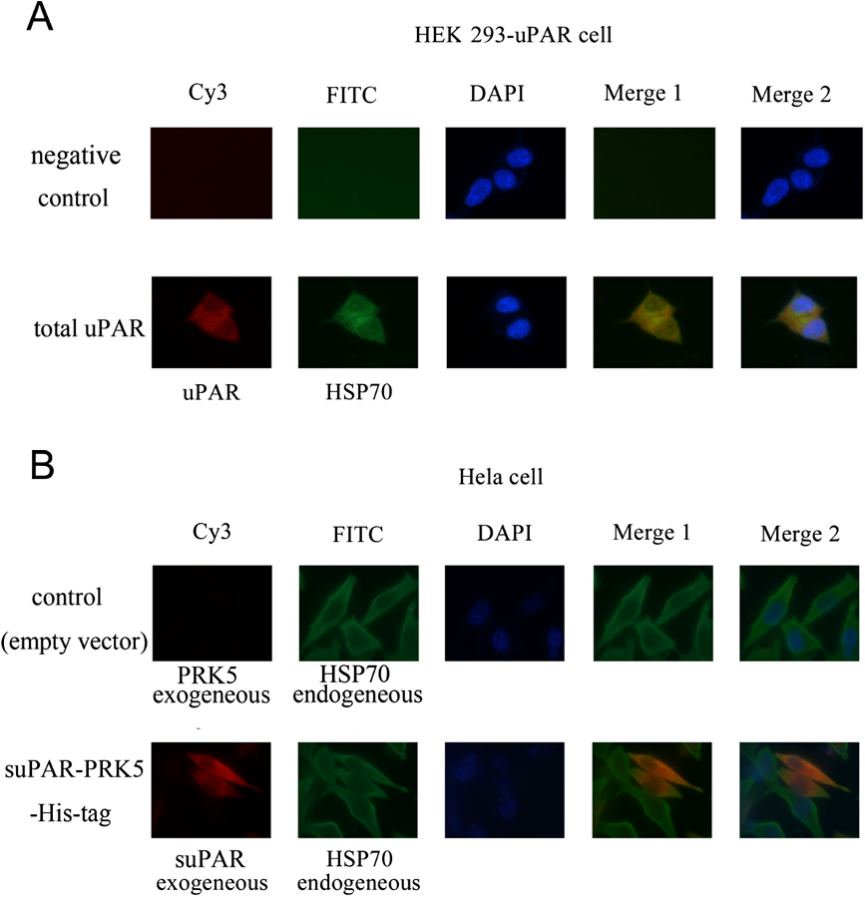
**Co-localization of HSP70 with uPAR in cytoplasm. (A)** uPAR and HSP70 co-localized in cytoplasm of HEK293-uPAR cells. Cells were fixed and then permeabilized with 0.5% Triton X-100 to show the total uPAR localization (down lane). The cells were then immune-stained with 1% BSA as a negative control (top lane) or with anti-uPAR antibody (in red) and anti-HSP70 antibody (in green) and stained with Alexa Fluor 488 (uPAR) and Alexa Fluor 594 (HSP70) conjugated secondary antibodies at room temperature. Co-localization appeared in yellow color. **(B)** Co-locolization of exogenous soluble uPAR and endogenous HSP70 in Hela cells. Cells transfected with PRK5 vector (upper lane) or soluble uPAR-His-tag (lower lane) were fixed and then permeabilized with 0.5% Triton. The cells were then immunostained with anti-His-tag antibody (in red) and anti-HSP70 antibody (in green) and stained with Alexa Fluor 488 (uPAR) and Alexa Fluor 594 (HSP70) conjugated secondary antibodies at room temperature, co-localization appeared in yellow color. Data shown are representative of three independent experiments.

We next examined whether the association between uPAR and HSP70 resulted in co-localization of the two proteins using double immunofluorescence staining in the HEK 293-uPAR cells. As shown in Figure 4A, uPAR and HSP70 co-localized throughout the cytoplasm in exponentially growing HEK 293-uPAR cells. To determine the cytoplasm distribution of uPAR and HSP70, a fluorescent nucleistain-DAPI that is only present in the nucleolus, was used together with the anti-uPAR and anti-HSP70 antibodies. As shown in Figure 4A, cells stained with secondary antibodies alone showed corresponding nuclear (DAPI) staining only (Blue) (up panel) suggesting the specificity of the indicated antibodies. In addition, we studied the intracellular distribution of soluble uPAR (His-tag suPAR) to determine whether HSP70 can also co-localize with suPAR. The data indicated that there was a significant overlap between exogenous suPAR and endogenous HSP70 in Hela cells as showed in Figure 4B. The association of suPAR and HSP70 was also observed in HEK293T and HEK 293-uPAR cells (data not shown), as the extent of overlapping differed from cell to cell, suggesting the dynamic feature of the interaction between suPAR and HSP70. Collectively, our data demonstrated that uPAR co-localized with HSP70 in the cytoplasm.

### Knockdown of HSP70 and MRJ inhibited uPAR-mediated cell adhesion in HEK 293-uPAR cells

**Figure 5:**
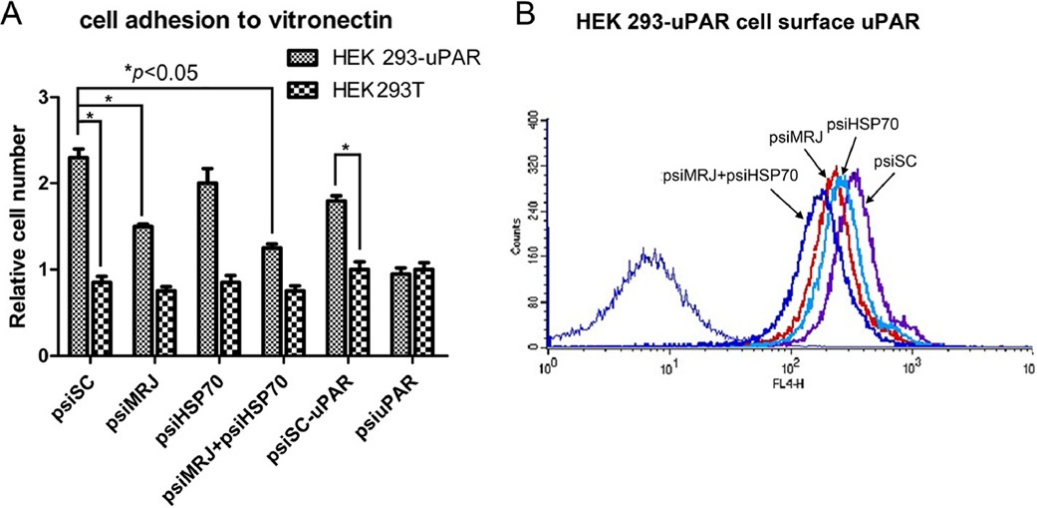
**Decrease of uPAR-mediated adhesion to vitronectin by knockdown of MRJ and/or HSP70 in HEK293-uPAR and HEK293T cells. (A)** Adhesion assay. The cells were transfected with psiSC, or psiMRJ, or psiHSP70 or psiMRJ plus psiHSP70 and grown on 96-well plates pre-coated with vitronectin (1 μg/ml) and stained with Giemsa and then quantified using Safire Fluorescence Absorbance and Luminescence Reader (TECAN). *P < 0.05: significantly different from the control. The experiments were repeated twice and performed in triplicate. **(B)** Reduction of uPAR on the cell surface by knockdown of MRJ/HSP70 using flow cytometry in HEK293-uPAR cells. The cells were transfected with psiSC, or psiMRJ, or psiHSP70, or psiMRJ plus psiHSP70. Forty-eight hours after transfection, cells were harvested and labeled with anti-human uPAR monoclonal antibody for 30 min. After labeling, the cells were immediately analysed by flow cytometry in APC channel. Suitable negative isotype controls were used to rule out the background fluorescence.

To test the biological significance of the uPAR-MRJ-HSP70 interaction, we next examined whether HSP70 and/or MRJ regulated uPAR-dependent adhesion to the ligand vitronectin in cells. We have previously reported that MRJ regulates uPAR-dependent adhesion to vitronectin and uPAR is involved in adhesion of HEK293 cells stably transfected with uPAR to vitronectin [23]. As shown in Figure 5A, psiMRJ or psiHSP70 led to a 30% reduction and 10% reduction respectively in HEK 293-uPAR cells adhesion to vitronectin, while psiHSP70 and psiMRJ in combination caused a 50% reduction. However, all these siRNA plasmids alone or in combination had no obvious effect in adhesion of uPAR negative HEK293T cells to vitronectin, suggesting that the inhibition effects by knockdown of HSP70 and/or MRJ were mediated by uPAR. To confirm this, flow cytometry was performed to determine the cell surface uPAR level after knockdown of HSP70 and/or MRJ. As shown in Figure 5B, suppression of MRJ and HSP70 simultaneously exhibited significant decrease cell surface uPAR, while knockdown of HSP70 or MRJ alone had only weak inhibition for cell surface uPAR. The decreased cell surface uPAR by knockdown of MRJ and/or HSP70 may account for reduced cell adhesion and further confirmed the interaction between uPAR, HSP70 and MRJ.

### HSP70 and MRJ siRNAs suppressed cell invasion and migration

**Figure 6:**
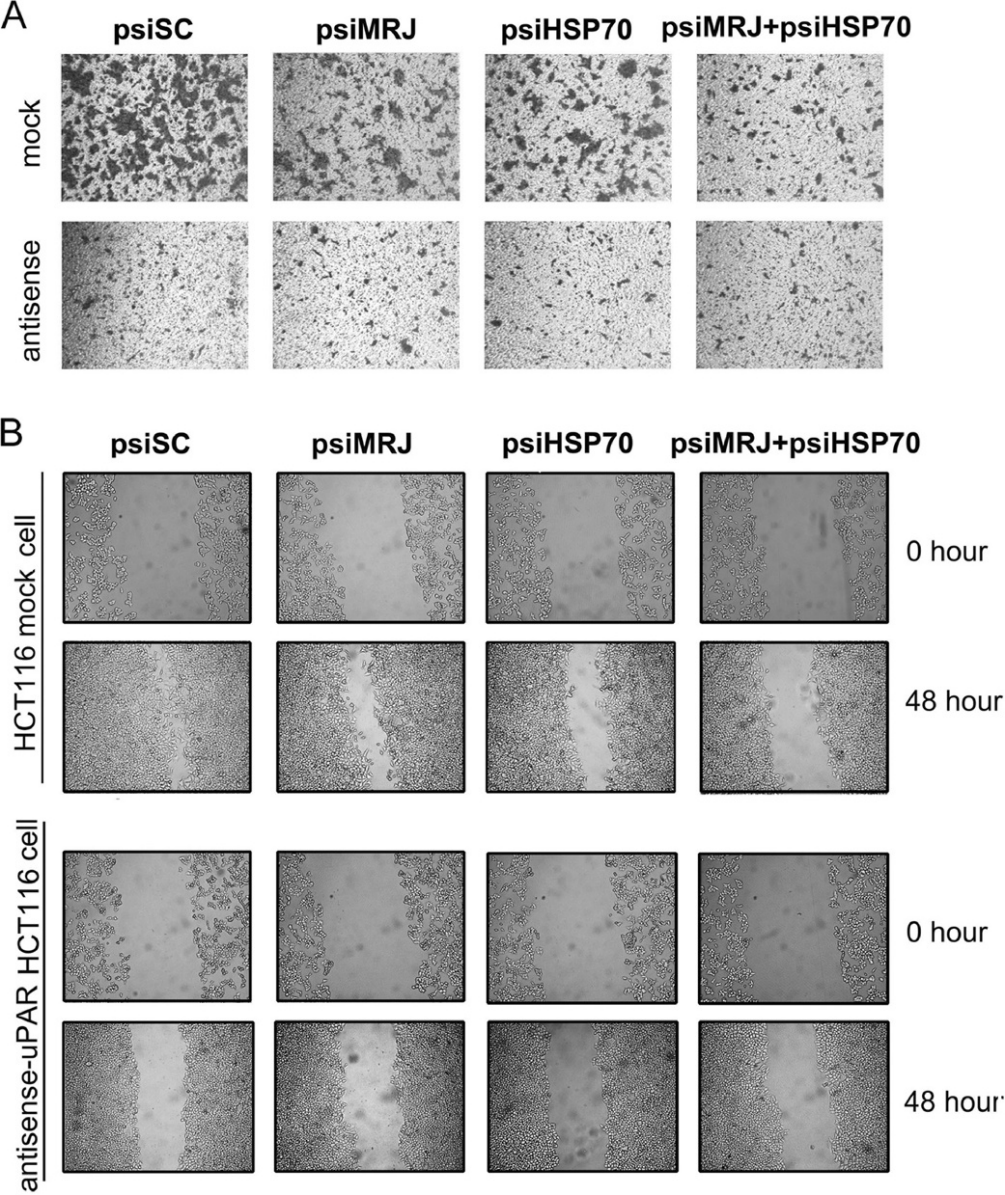
**Invasion and migration assays in HCT116 mock cells and antisense-uPAR HCT116 cells by knockdown of HSP70 and/or MRJ. (A)** Transwell assays. Cells were transfected with psiSC, or psiMRJ, or psiHSP70, or psiMRJ plus psiHSP70 and starved for 12 hours then applied to the upper compartment of the transwell chamber. HCT116 mock cells (upper lane) and antisense-uPAR HCT116 cells (lower lane) migrated to the lower surface of the membrane were fixed and stained with 0.1% crystal violet. Representative images were shown. The experiments were repeated for three times. **(B)** Wound healing assays. HCT116 mock cells (upper lanes) and antisense-uPAR HCT116 cells (lower lanes) were transfected with psiSC, or psiMRJ, or psiHSP70, or psiMRJ plus psiHSP70. Photographs were taken immediately and after 48 hours of creating the scratch. Images shown are representative of three independent experiments.

Previous reports have verified the essential roles of uPAR in tumor invasion and metastasis [29, 30]. To determine the role of HSP70/MRJ in cell invasion and migration, we conducted transwell and wound healing assays. In these experiments we used a pair of cell lines, HCT116 mock cells transfected with vector only that expresses high levels of uPAR and antisense-uPAR HCT116 cells that expresses low levels of uPAR. Transwell assay (Figure 6A) showed that cells transfected with psiHSP70 and psiMRJ alone exhibited a mild inhibition in the cell invasiveness, and combination of psiHSP70 and psiMRJ led to enhanced inhibition in HCT116 mock cells. However, no migration changes were evident in the antisense-uPAR HCT116 cells transfected with psiHSP70 and/or psiMRJ.

Wound healing scratch assay was also performed to examine whether down-regulation of HSP70 and MRJ could inhibit HCT116 cell migration. As shown in Figure 6B, 48 hours after the scratch, HCT116 mock cells transfected with psiSC migrated into and largely covered the original wound area, whereas those transfected with psiMRJ and/or psiHSP70 failed to cover a substantial portion of the wound. The results revealed that psiMRJ and psiHSP70 in combination led to enhanced inhibition of cell migration as compared to psiMRJ or psiHSP70 alone. However, the suppression of migration in the antisense-uPAR HCT116cells was not as significant as in HCT116 mock cells suggesting that the HSP70/MRJ suppressed migration at least partially through the inhibition of uPAR. Moreover, the cytoskeleton protein F-actin in HCT116 mock cells transfected with psiHSP70 or psiMRJ were redistributed when compared to control cells transfected with psiSC (Additional file 1). Taken together, these data suggest strongly that the interaction between HSP70/MRJ and uPAR play a role in regulating the migration of HCT116 cells *in vitro*. Furthermore we found that siRNA-mediated knockdown of HSP70 or/and MRJ had no obvious effects on cell proliferation and cell cycle in above cells (Additional file 2).

### Knockdown of HSP70/MRJ reduced expression of invasion related genes mmp2 and mmp9

**Figure 7:**
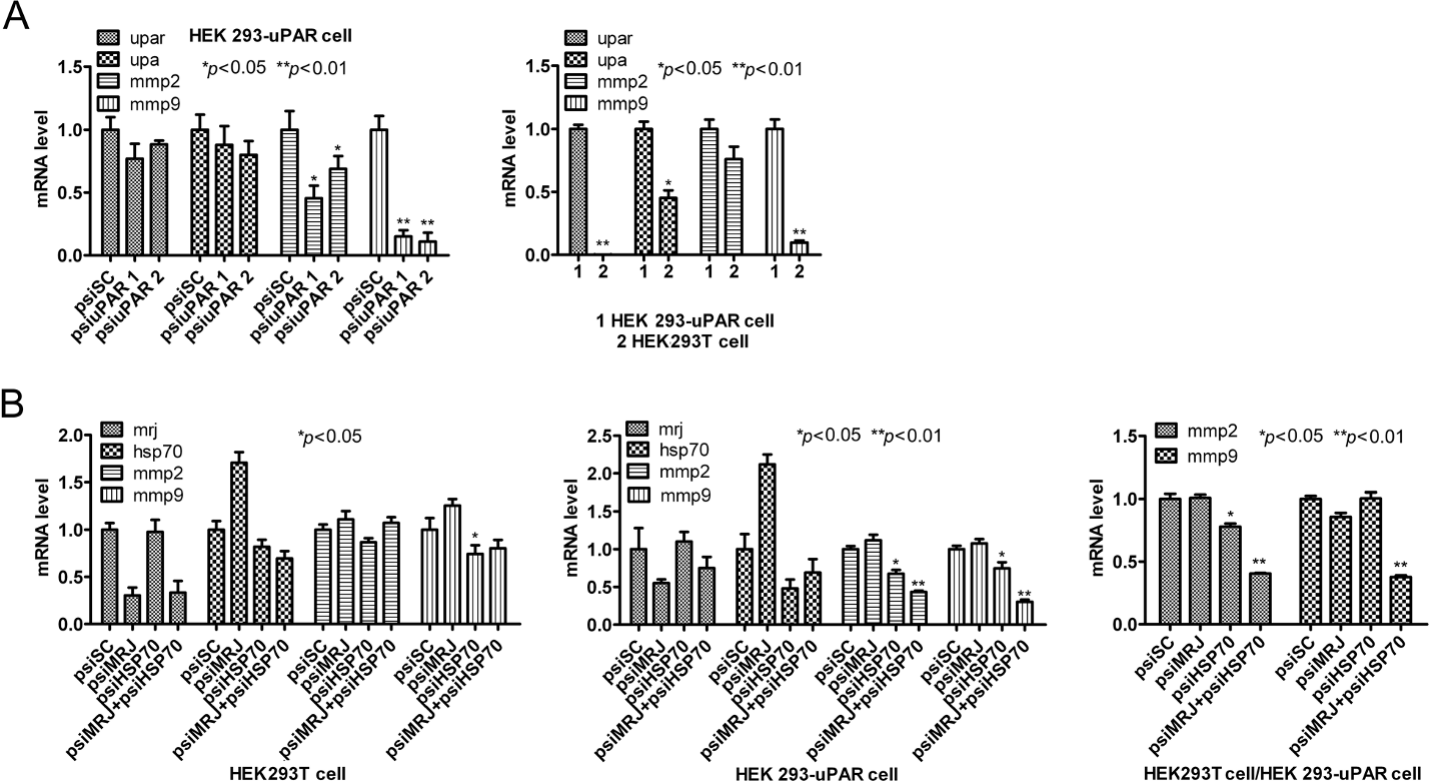
**Knockdown of HSP70/MRJ alters the expression of invasion related genes mmp2 and mmp9 using quantitative RT-PCR analysis. (A)** Knockdown of uPAR decreased the mRNA levels of mmp2 and mmp9. Total RNAs were isolated from a pair of stably transfected cell lines (HEK 293-uPAR vs HEK293T cells) and from HEK 293-uPAR cells transiently transfected with psiuPAR or psiSC for 48 hours. The relative levels of the candidate genes (upar, upa, mmp2 and mmp9) were analyzed by quantitative RT-PCR (Q-PCR) analysis. **(B)** Knockdown of MRJ/HSP70 decreased mRNA levels of mmp2 and mmp9. Total RNAs were isolated from HEK 293-uPAR or HEK293T cells after transfected with psiSC, or psiMRJ, or psiHSP70, or psiMRJ plus psiHSP70 for 48 hours. The relative levels of the candidate genes (mrj, hsp70, upar, upa, mmp2 and mmp9) were analyzed by quantitative-PCR. The comprehensive inhibition ratio was calculated from the results of the mRNA level in HEK 293-uPAR cells divided by the mRNA level in HEK293T cells. The results represent three independent experiments with triplicate samples for each experiment *P < 0.05: significant different from the control.

It is documented that the uPAR initiates ECM enzyme degradation to promote extracellular proteolysis [1, 3]. The uPA/uPAR system induces MMP activity and promotes cancer cell invasion and metastasis. Previous reports suggested that down-regulation of uPAR decreased the expression of mmp2 and mmp9 [31], which was consistent with our Q-PCR results as shown in Figure 7A. The levels of mmp2 and mmp9 mRNAs were reduced about 50% and 90% respectively in HEK 293-uPAR cells transfected with psiuPAR (Figure 7A, left lane). To study whether inhibition of uPAR reduces expression of mmp2 and mmp9 genes, a pair of cell lines used in this paper were used (Figure 7A, right lane). In HEK 293-uPAR cells which express high amounts of uPAR, mmp2 and mmp9 mRNAs were over-expressed, while in HEK293T cells, mmp2 and mmp9 mRNAs were less-expressed. To study whether knockdown of HSP70/MRJ reduces expression of mmp2 and mmp9 genes, the HEK 293-uPAR cells were transfected with psiSC, or psiMRJ, or psiHSP70, or psiHSP70 plus psiMRJ. The HEK293T cells transfected with the above siRNAs were served as controls. As shown in Figure 7B, down-regulation of both HSP70 and MRJ expression in HEK 293-uPAR cells (Figure 7B, middle lane) caused more significant reduction of mmp2 and mmp9 mRNAs compared with that in HEK293T cells (Figure 7B, left lane). To eliminate the background and to determine effect of uPAR, comprehensive inhibition ratios were calculated with the results of the gene expression abundances in HEK 293-uPAR cells divided by the abundance in HEK293T cells (Figure 7B, right lane). The results suggested that the inhibition effects were mainly via uPAR. Collectively, these findings indicated that the effects of HSP70/MRJ on mmp2 and mmp9 gene expression in HEK 293-uPAR/HEK293T cells may be mediated by uPAR through uPAR/HSP70/MRJ interactions. Moreover, these data further indirectly confirm that the association of uPAR/HSP70/MRJ plays a critical role in cell migration and invasion.

### Over-expression of HSP70 and MRJ increased phosphorylation of FAK, ERK1/2and AKT

**Figure 8:**
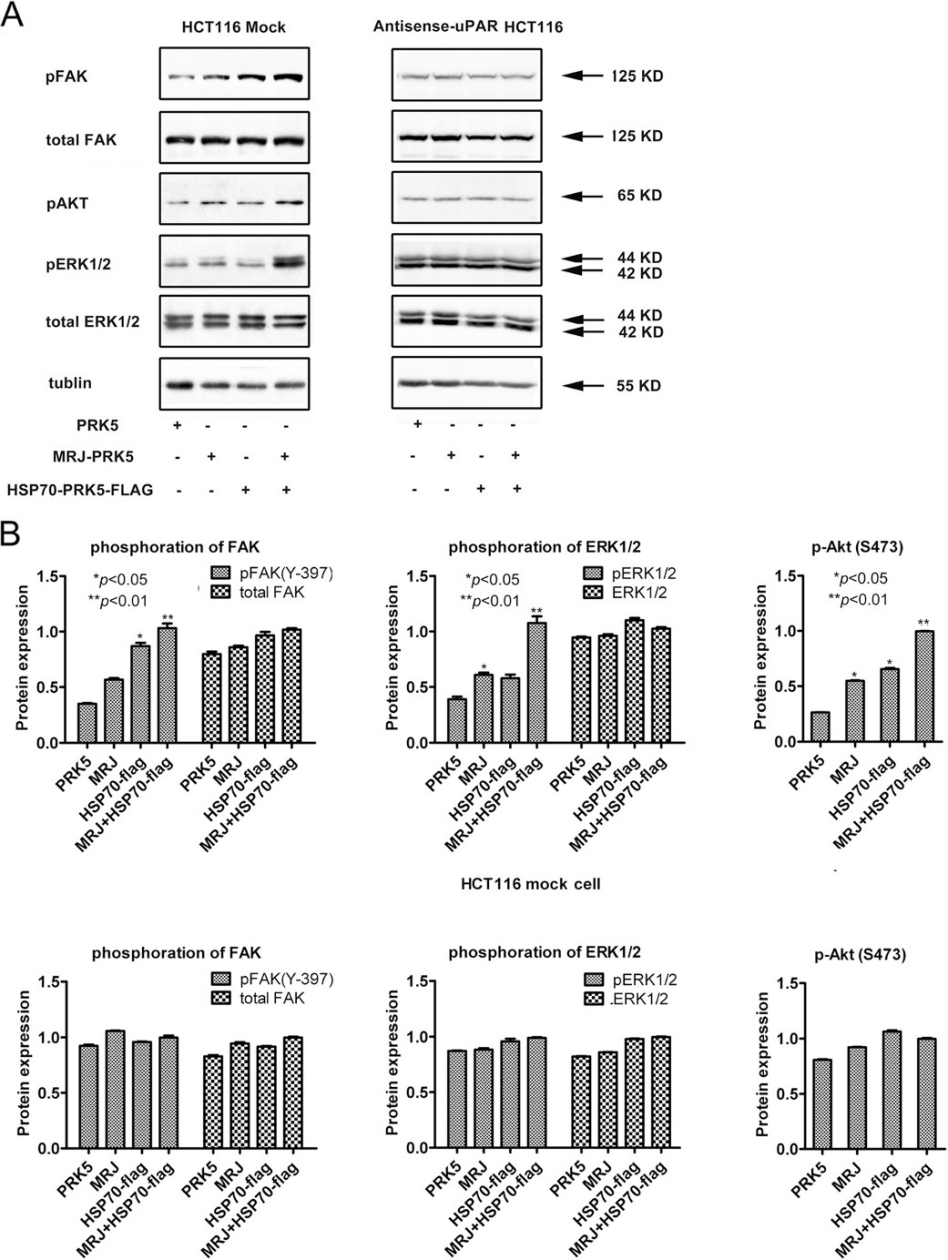
**Phosphorylation of ERK1/2, FAK and AKT by over-expression of HSP70/MRJ is mediated by uPAR in HCT116 cells. (A)** Cells were transfected with PRK5, or MRJ-PRK5, or HSP70-PRK5-flag or MRJ-PRK5 plus HSP70-PRK5-flag as shown in the figure. Forty-eight hours after transfection, cells were lysed and 60 μg of total protein from each sample was loaded on SDS-PAGE. Then, the protein levels of ERK1/2, p-ERK1/2, FAK, p-FAK and p-AKT were analyzed by Western blot. Tubulin was used as a loading control. The results shown are representative of three independent experiments. **(B)** The bands shown in **(A)** were scanned by densitometry using Image J software. The ratios of phopho-ERK vs ERK, phospho-FAK vs FAK and p-AKT were calculated. The ratios in MRJ plus HSP70-transfected cells were arbitrarily taken as 1.0 and values of all others relative to it were calculated accordingly. The histagram shows the mean ± SD, *P < 0.05: significantly different from the control, *P < 0.01: highly significantly different from the control.

Binding of uPA to uPAR on the cell surface activates FAK, c-Src, H-Ras, AKT [32], extracellular signal-regulated kinase (ERK)/MAPK [33] and myosin light chain kinase [34]. uPAR binds directly to vitronectin and promotes activation of Rac1 [35]. These uPAR-dependent cell signaling events impact cell migration and survival. To determinate the effect of interaction between HSP70/MRJ and uPAR on uPAR-mediated intracellular signaling, we examined the signal proteins in cells over-expressing HSP70 and/or MRJ. The results indicated that in HCT116 mock cells, HSP70 or MRJ increased phosphorylation of FAK, ERK1/2 and AKT significantly (*P* < 0.05), while total FAK and ERK1/2 levels remained unchanged (Figure 8A, left lane). In contrast, in antisense-uPAR HCT116 cells, HSP70 or MRJ had no effect (Figure 8A, right lane), suggesting that HSP70 or MRJ activated these signal pathways through the interaction with uPAR. These data reinforce the association of HSP70/MRJ with uPAR and may explain mechanisms involved in the uPAR/HSP70/MRJ complex-mediated cell adhesion, invasion and migration [36].

## Discussion

Although uPAR and HSP70/MRJ are separately known to be involved in cell metabolism, physical, functional interaction of them resulting in changes of cell function have not been reported previously. The MRJ has been identified as an interacting protein of uPAR in our previous report. In this study, we report for the first time that HSP70, MRJ and uPAR form a triple complex and interact with one another, resulting in enhanced uPAR-dependent invasion, migration and adhesion to vitronectin.

The uPAR is an important regulator of several cellular processes including proliferation, migration, adhesion, as well as cell signaling. Similar to uPAR, over-expression of the HSP70 and MRJ increases tumor growth, cancer cell migration and metastatic potential. Recent findings indicate that cancer cells express high levels of HSPs which are closely correlated with poor prognosis [26]. MRJ is an essential co-chaperone of HSP70, with the N-terminal J-domain necessary for its interaction with HSP70 and its chaperone activity [24, 25]. In breast cancer, similar to uPAR, HSP70 expression is correlated with metastasis and poor prognosis [25, 26]. Under non-stress conditions, the HSP70 and MRJ have multiple housekeeping functions including folding and trans-locating newly synthesized proteins, as well as activating signaling molecules.

In our study uPAR was found to bind specifically to HSP70 but not HSP27 in HCT116 cells using co-IP analysis (data not shown). The HSP27 is a small heat shock protein which expresses high levels in many types of cancer cells [37]. Similarly the uPAR specifically bound to MRJ (DNAJB6), but not to DNAJB4 (another MRJ family member) in a breast cancer cell line MDA-MB-231 by using co-IP assay. These data suggest that not all heat shock proteins can interact with uPAR and the interactions between uPAR and HSP70/MRJ are specific. These experiments were just additional controls to the standard IgG controls to determine specificity of the interaction between uPAR and HSP70 and MRJ. The co-IPs of uPAR and HSP70/MRJ in wild-type HCT116 cells indicate that these proteins interact with each other when they expressed endogenously.

Our studies found that over-expression of MRJ or HSP70 enhanced the interaction between HSP70 and MRJ and uPAR, however knockdown of MRJ or HSP70 reduced the formation of the triple complex. The fact that MRJ regulates the formation of uPAR-HSP70 complex suggests that MRJ may act as an uPAR-specific adaptor to link uPAR to the HSP70 protein. Furthermore, knockdown of HSP70 and/or MRJ also decreased cell-cytosol uPAR protein level in HCT116 cells and cell-surface uPAR protein level in HEK 293-uPAR cells. Further study suggested that uPAR may be a possible client protein of HSP70 and MRJ, and knockdown of HSP70 or MRJ may lead to the degradation of the client protein by the proteasome. Previous reports showed that when the uPAR/uPA/PAI-1 complex associates with α2MR-LRP the entire complex is redistributed to clathrin coated vesicles for internalization, after which uPA and PAI-1 are degraded in the lysosome [38–40], but the internalized uPAR is recycled to the cell surface from the cytosol. MRJ is expressed predominantly in the cytoplasm, but HSP70 can interact with lipids and transport to cell membrane [41]. We thus speculate that HSP70 and MRJ form a complex and interact with uPAR in the cytoplasm and then HSP70 helps to recycle uPAR to the cell surface. It is still not clear whether the interaction between HSP70, MRJ and uPAR is taking place during uPAR folding, protein maturation and transportation to the cell surface and/or occurs after uPAR/uPA/PAI-1 internalization and uPAR recycling.

As shown in Figure 5A, the important uPAR/vitronectin interaction is at least in part regulated by HSP70 and/or MRJ. uPAR-dependent cell adhesion to the ECM protein vitronectin is an important event in wound healing, tissue remodeling, immune response and cancer development. Previous reports showed that on the surface of endothelial and U937 cells, uPAR can mediate cell adhesion to vitronectin [42, 43]. The interaction between vitronectin and uPAR has also been implicated in regulating processes necessary for endothelial cell invasion and migration at vitronectin-rich extracellular matrix sites as well as facilitating intracellular signaling [42]. Functionally the interaction between uPAR and vitronectin can promote both cellular adhesion and migration [42–44] and may direct uPAR to focal contacts [8, 45]. In HEK 293-uPAR cells, suppression of HSP70 or/and MRJ reduced uPAR-mediated cell adhesion to vitronectin significantly. We assume that MRJ and/or HSP70 knockdown may decrease cell-surface uPAR resulting in less interaction between uPAR and vitronectin. However, the exact mechanism how MRJ/HSP70 regulates uPAR-mediated cell adhesion to vitronectin is still not clear and need more study.

It is known that migrating cells must break down the established cell adhesion sites to detach from its substrate and establish new contact points between a cell and its underlying substrate to provide the necessary adhesion that a cell needs to move forward. Therefore, regulation of HSP70/MRJ on cell adhesion may play important roles in cell motility. As shown in this paper knockdown of HSP70 and/or MRJ inhibited cell migration by using wound healing and transwell assays in HCT116 cells. Further experiments indicated that knockdown of HSP70 or MRJ had an effect on cytoskeleton reorganization. Recent studies have shown that the HSP70 can participate in cell migration by helping to target key regulatory proteins such as tissue transglutaminase to the leading edges of migrating cells [46]. Our results thus indicated that the interactions between HSP70, MRJ and uPAR can promote cell invasion and migration.

uPA-uPAR binding promotes cleaving plasminogen to plasmin which cleaves and activates matrix metalloproteases (MMPs) to enhance cell invasion, suggesting a mechanism that reducing cell-surface uPAR will decrease the expression of mmp2 and mmp9, resulting in total abrogation of matrix degradation. It is reported that inhibition of extracellular HSP70 and HSP90 reduce MMP-2 activation and decrease cancer cell migration and invasion [47, 48]. This and our data demonstrate that uPAR and HSP70/MRJ complex may promote matrix degradation, as knockdown of HSP70 and/or MRJ decreased the mRNA levels of mmp2 and mmp9, two important cancer invasion related genes.

Furthermore, cell signaling pathways, such as MEK1/2-ERK1/2 and PI3K-AKT, are shown to be downstream responses of uPAR activation. In this paper, we demonstrated that over-expression of HSP70 and/or MRJ enhanced phosphorylated signals of ERK, JNK, and AKT. Activation of these signaling pathways was correlated with significant increases in migration and invasion capacities when HSP70 and/or MRJ were over-expressed. Thus, we favor a model in which HSP70 or MRJ-induced uPAR expression activates cell signaling pathways that are complementary in inducing the full spectrum of cellular changes observed in adhesion and migration [48].

## Conclusion

In conclusion, our findings suggest a novel function of HSP70/MRJ/uPAR complex in cell adhesion, invasion and migration, and may provide more understanding in the mechanisms of uPAR-mediated cancer metastasis. The finding of triple complex interaction and its biological significance may promote basic research and further highlight the uPAR as a molecular target for therapy.

## Electronic supplementary material

Additional file 1: **Knockdown of HSP70/MRJ modulates cytoskeleton reorganization.** HCT116 mock cells were transfected with psiHSP70 or psiMRJ. F-actin was visualized with Texas Red-X phalloidin. Scale bar = 20 μm. (PDF 148 KB)

Additional file 2: **Knockdown of HSP70/MRJ has no obvious effects on cell proliferation and cell cycle.** (A) HCT116 mock cells and antisense-uPAR HCT116 cells were transfected with psiHSP70 and/or psiMRJ. Twenty-four hours later, all cells were harvested for flow cytometry analysis. The experiments were carried out independently in triplicate. (B) HCT116 mock cells and antisense-uPAR HCT116 cells were transfected with psiHSP70 and/or psiMRJ, and the cell viability was assessed by MTT assay. (C) MCF-7 and MB-MDA-231 cells were transfected with psiHSP70 and/or MRJ. Twenty-four hours later, cells were fixed and stained with PI for flow cytometry as described in Materials and Methods. DNA histograms were modeled with CellQuest analysis software. Phase percentages for G0/G1, S, and G2/M are depicted by bar graph. Data represent mean values of triplicate samples. (PDF 223 KB)

## Abbreviations

ECM: Extra cellular matrix
GPI: Glycosyl phosphatidylinositol
HSP: Heat shock protein
MRJ: Mammalian relative of DnaJ
PBS: Phosphate-buffered saline
SDS: Sodium dodecyl sulfate
uPA: Urokinase-type plasminogen activator
uPAR: uPA receptor.
