# Supplementary material for: Heat shock proteins HSP70 and MRJ cooperatively regulate cell adhesion and migration through urokinase receptor
Source: BMC Cancer. 2014 Aug 30;14:639. doi: 10.1186/1471-2407-14-639 (PMC4159539; doi:10.1186/1471-2407-14-639)

Additional file 1: Knockdown of HSP70/MRJ modulates cytoskeleton reorganization. HCT116 mock cells were transfected with psiHSP70 or psiMRJ. F-actin was visualized with Texas Red-X phalloidin. Scale bar = 20 μm.

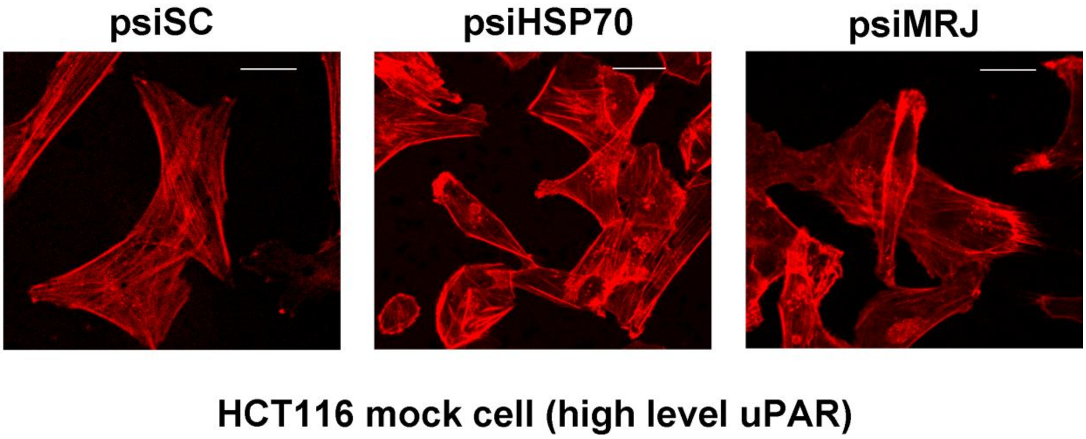

Supplement: Supplementary file 1 — Additional file 1: Knockdown of HSP70/MRJ modulates cytoskeleton reorganization. HCT116 mock cells were transfected with psiHSP70 or psiMRJ. F-actin was visualized with Texas Red-X phalloidin. Scale bar = 20 μm. (PDF 148 KB) [file 12885_2013_4811_MOESM1_ESM.pdf]
