# Supplementary material for: Heat shock proteins HSP70 and MRJ cooperatively regulate cell adhesion and migration through urokinase receptor
Source: BMC Cancer. 2014 Aug 30;14:639. doi: 10.1186/1471-2407-14-639 (PMC4159539; doi:10.1186/1471-2407-14-639)
Supplement: Supplementary file 2 — Additional file 2: Knockdown of HSP70/MRJ has no obvious effects on cell proliferation and cell cycle. (A) HCT116 mock cells and antisense-uPAR HCT116 cells were transfected with psiHSP70 and/or psiMRJ. Twenty-four hours later, all cells were harvested for flow cytometry analysis. The experiments were carried out independently in triplicate. (B) HCT116 mock cells and antisense-uPAR HCT116 cells were transfected with psiHSP70 and/or psiMRJ, and the cell viability was assessed by MTT assay. (C) MCF-7 and MB-MDA-231 cells were transfected with psiHSP70 and/or MRJ. Twenty-four hours later, cells were fixed and stained with PI for flow cytometry as described in Materials and Methods. DNA histograms were modeled with CellQuest analysis software. Phase percentages for G0/G1, S, and G2/M are depicted by bar graph. Data represent mean values of triplicate samples. (PDF 223 KB) [file 12885_2013_4811_MOESM2_ESM.pdf]

Additional file 2: Knockdown of HSP70/MRJ has no obvious effects on cell proliferation and cell cycle. (A) HCT116 mock cells and antisense-uPAR HCT116 cells were transfected with psiHSP70 and/or psiMRJ. Twenty-four hours later, all cells were harvested for flow cytometry analysis. The experiments were carried out independently in triplicate. **(B)** HCT116 mock cells and antisense-uPAR HCT116 cells were transfected with psiHSP70 and/or psiMRJ, and the cell viability was assessed by MTT assay. **(C)** MCF-7 and MB-MDA-231 cells were transfected with psiHSP70 and/or MRJ. Twenty-four hours later, cells were fixed and stained with PI for flow cytometry as described in Materials and Methods. DNA histograms were modeled with CellQuest analysis software. Phase percentages for G0/G1, S, and G2/M are depicted by bar graph. Data represent mean values of triplicate samples.

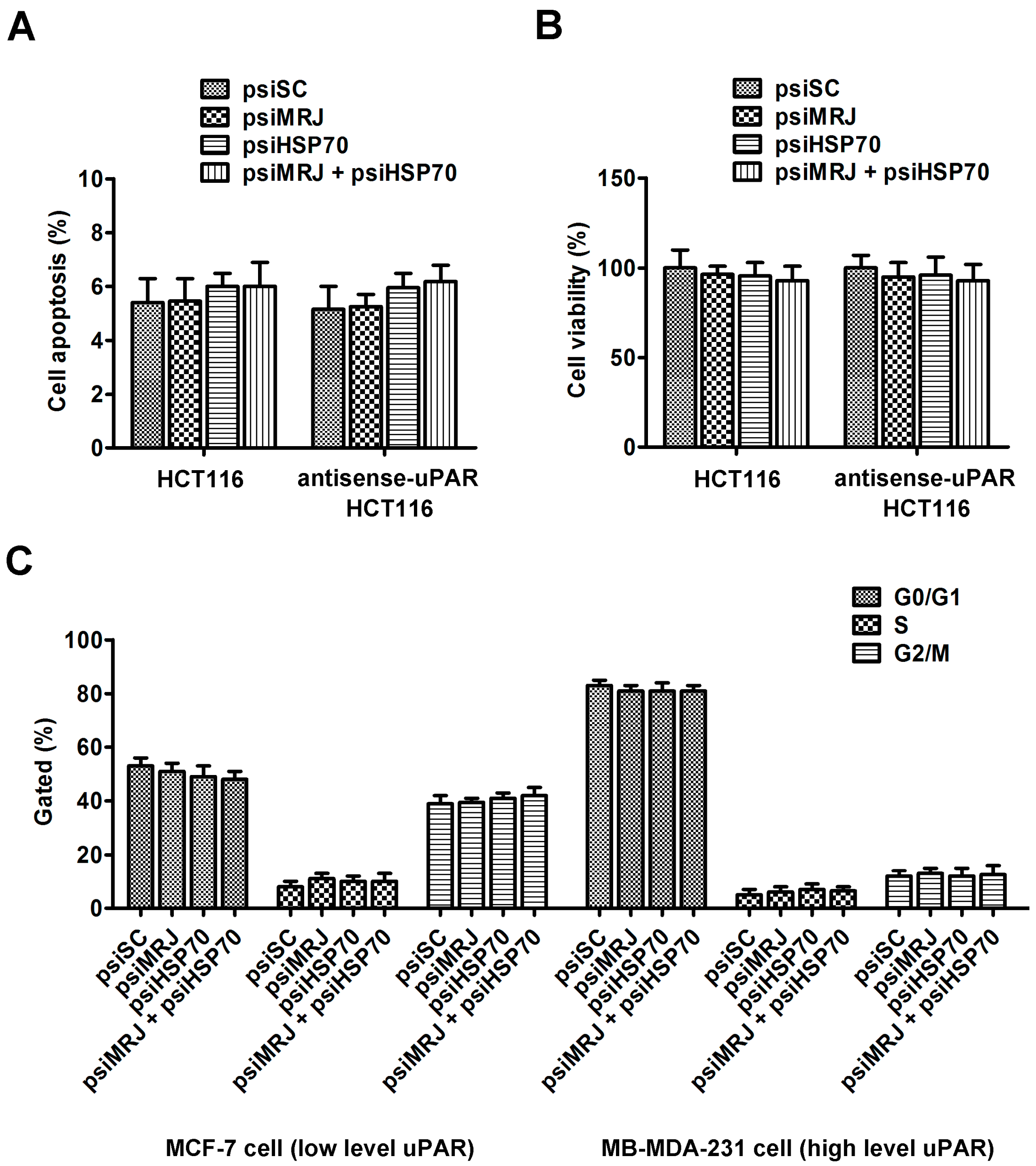
A
psiSC
psiMRJ
psiHSP70
psiMRJ + psiHSP70
Cell apoptosis (%)
0
2
4
6
8
10
HCT116
antisense-uPAR HCT116
B
psiSC
psiMRJ
psiHSP70
psiMRJ + psiHSP70
Cell viability (%)
0
50
100
150
HCT116
antisense-uPAR HCT116
C
G0/G1
S
G2/M
Gated (%)
0
20
40
60
80
100
psiSC
psiMRJ
psiHSP70
psiMRJ + psiHSP70
MCF-7 cell (low level uPAR)
MB-MDA-231 cell (high level uPAR)
